# Supplementary material for: Control of mitophagy initiation and progression by the TBK1 adaptors NAP1 and SINTBAD
Source: Nat Struct Mol Biol. 2024 Jun 25;31(11):1717–31. doi: 10.1038/s41594-024-01338-y (PMC11564117; doi:10.1038/s41594-024-01338-y)
Supplement: Supplementary file 2 — Reporting Summary [file 41594_2024_1338_MOESM2_ESM.pdf]

nature portfolio

Corresponding author(s): Elias Adriaenssens
Michael Lazarou
Sascha Martens

Last updated by author(s): 26/4/24

Reporting Summary

Nature Portfolio wishes to improve the reproducibility of the work that we publish. This form provides structure for consistency and transparency in reporting. For further information on Nature Portfolio policies, see our Editorial Policies and the Editorial Policy Checklist.

Statistics

For all statistical analyses, confirm that the following items are present in the figure legend, table legend, main text, or Methods section.

n/a	Confirmed	
☐	☒	The exact sample size (*n*) for each experimental group/condition, given as a discrete number and unit of measurement
☐	☒	A statement on whether measurements were taken from distinct samples or whether the same sample was measured repeatedly
☐	☒	The statistical test(s) used AND whether they are one- or two-sided<br>*Only common tests should be described solely by name; describe more complex techniques in the Methods section.*
☒	☐	A description of all covariates tested
☐	☒	A description of any assumptions or corrections, such as tests of normality and adjustment for multiple comparisons
☐	☒	A full description of the statistical parameters including central tendency (e.g. means) or other basic estimates (e.g. regression coefficient) AND variation (e.g. standard deviation) or associated estimates of uncertainty (e.g. confidence intervals)
☐	☒	For null hypothesis testing, the test statistic (e.g. *F*, *t*, *r*) with confidence intervals, effect sizes, degrees of freedom and *P* value noted<br>*Give P values as exact values whenever suitable.*
☒	☐	For Bayesian analysis, information on the choice of priors and Markov chain Monte Carlo settings
☒	☐	For hierarchical and complex designs, identification of the appropriate level for tests and full reporting of outcomes
☒	☐	Estimates of effect sizes (e.g. Cohen's *d*, Pearson's *r*), indicating how they were calculated

Our web collection on statistics for biologists contains articles on many of the points above.

Software and code

Policy information about availability of computer code

Data collection	Confocal microscopy images were collected using ZEN software version 2022 (Carl Zeiss Microscopy, GmbH, Germany) connected to a LSM700 or an inverted Leica SP8 confocal laser scanning microscope and the Leica Application Suite X (LASX v2.0.1).
Data analysis	1. FlowJo software (Version 10.9.0; Tree Star Inc., Ashland, OR, USA) for FACS-data analysis (RRID:SCR_008520).<br>2. PRISM software (Version 9.5.1; Graphpad Software, La Jolla, CA, USA) for statistical analysis and generating graphs.<br>3. ImageJ software (Schindelin et al. 2015) (version 2.14.0/1.54f) for immunofluorescence microscopy image analysis (RRID:SCR_003070).<br>4. FACSDiva software (BD FACSDiva software) for flow cytometry experiments (RRID:SCR_001456).<br>5. Synthego ICE v2 CRISPR Analysis tool (Synthego) for CRISPR knockout experiments.<br>6. FreeStyle 1.7 software (Thermo Scientific) for mass spectrometry data.<br>7. MaxQuant software version 1.6.17.0 for mass spectrometry data.<br>8. Leica Application Suite X (LASX v2.0.1) (RRID:SCR_013673)

For manuscripts utilizing custom algorithms or software that are central to the research but not yet described in published literature, software must be made available to editors and reviewers. We strongly encourage code deposition in a community repository (e.g. GitHub). See the Nature Portfolio guidelines for submitting code & software for further information.

April 2023

Data

Policy information about availability of data

All manuscripts must include a data availability statement. This statement should provide the following information, where applicable:

- Accession codes, unique identifiers, or web links for publicly available datasets
- A description of any restrictions on data availability
- For clinical datasets or third party data, please ensure that the statement adheres to our policy

Raw files associated with this work are available on Zenodo (https://doi.org/10.5281/zenodo.10637353). The mass spectrometry proteomics data have been deposited to the ProteomeXchange Consortium via the PRIDE partner repository [88] with the dataset identifier PXD049184. For screening CRISPR knockout clones we used Human Genome Variation Society (HGVS; http://varnomen.hgvs.org/). Mass spectrometry dataset was analyzed using Uniprot human proteome database (release 2021_03) and a database of common laboratory contaminants.

Research involving human participants, their data, or biological material

Policy information about studies with human participants or human data. See also policy information about sex, gender (identity/presentation), and sexual orientation and race, ethnicity and racism.

Reporting on sex and gender	N/A
Reporting on race, ethnicity, or other socially relevant groupings	N/A
Population characteristics	N/A
Recruitment	N/A
Ethics oversight	N/A

Note that full information on the approval of the study protocol must also be provided in the manuscript.

Field-specific reporting

Please select the one below that is the best fit for your research. If you are not sure, read the appropriate sections before making your selection.

☒ Life sciences ☐ Behavioural & social sciences ☐ Ecological, evolutionary & environmental sciences

For a reference copy of the document with all sections, see nature.com/documents/nr-reporting-summary-flat.pdf

Life sciences study design

All studies must disclose on these points even when the disclosure is negative.

Sample size	No statistical methods were applied to pre-evaluate sample size because of the exploratory nature of our study which entails that effect sizes were not known prior to the study. Experiments were performed at least as three replicates, according to current practices in the field. Statistical analysis was performed on experiments for which the sample size included at least 3 biological replicates. Sample sizes were based on previous experience and current standards in the field.
Data exclusions	No data were excluded from the analyses.
Replication	All experiments were replicated at least three times with similar findings. Samples sizes are provided in the figure legends.
Randomization	Samples were allocated into experimental groups by genotype of knockout condition. Covariates were controlled for by maintaining all samples in the same growth and media conditions.
Blinding	The investigators were not blinded to treatment or genotype allocations during this study. For cell based experiments, it was not possible to blind the experimenter as researchers needed to know experimental conditions for performing experiments.

Reporting for specific materials, systems and methods

We require information from authors about some types of materials, experimental systems and methods used in many studies. Here, indicate whether each material, system or method listed is relevant to your study. If you are not sure if a list item applies to your research, read the appropriate section before selecting a response.

Materials & experimental systems

n/a	Involved in the study
☐	☒ Antibodies
☐	☒ Eukaryotic cell lines
☒	☐ Palaeontology and archaeology
☒	☐ Animals and other organisms
☒	☐ Clinical data
☒	☐ Dual use research of concern
☒	☐ Plants

Methods

n/a	Involved in the study
☒	☐ ChIP-seq
☐	☒ Flow cytometry
☒	☐ MRI-based neuroimaging

Antibodies

Antibodies used

The primary antibodies used in this study for western blotting are:
anti-β-Actin (1:5000, Abcam Cat# ab20272, RRID:AB_445482)
anti- COXII (1:1000, Abcam Cat# ab110258, RRID:AB_10887758)
anti-COXII (1:1000, Cell Signaling Technology Cat# 31219, RRID:AB_2936222)
anti-FIP200 (1:1000, Cell Signaling Technology Cat# 12436, RRID:AB_2797913)
anti-HA (1:500, Cell Signaling Technology Cat# 2367, RRID:AB_10691311)
anti-HA (1:1000, Cell Signaling Technology Cat# 3724, RRID:AB_1549585)
anti-mHSP60 (1:1000, Abcam Cat# ab128567, RRID:AB_11145464)
anti-NAP1 (1:1000, Abcam Cat# ab192253, RRID:AB_2941051)
anti-OPTN (1:500, Sigma Aldrich Cat# HPA003279, RRID:AB_1079527)
anti-p62/SQSTM1 (1:1000, Abnova Cat# H00008878-M01, RRID:AB_437085)
anti-SINTBAD (1:1000, Cell Signaling Technology Cat# 8605, RRID:AB_10839270)
anti-TBK1 (1:1000, Cell Signaling Technology Cat# 38066, RRID:AB_2827657)
anti-TBK1 (1:1000, Cell Signaling Technology Cat# 3013, RRID:AB_2199749)
anti-phospho-TBK1 S172 (1:1000, Cell Signaling Technology Cat# 5483, RRID:AB_10693472)
anti-α-Tubulin (1:5000, Abcam Cat# ab7291, RRID:AB_2241126)
anti-PARKIN (1:200 Santa Cruz Biotechnology Cat# sc-32282, RRID:AB_628104)
anti-phospho-Ubiquitin S65 (1:2000, Millipore Cat# ABS1513-I, RRID:AB_2858191)
anti-ULK1 (1:1000, Cell Signaling Technology Cat# 8054, RRID:AB_11178668)

The secondary antibodies for western blotting used in this study are:
HRP conjugated polyclonal goat anti-mouse (Jackson ImmunoResearch Labs Cat# 115-035-003, RRID:AB_10015289)
HRP conjugated polyclonal goat anti-rabbit (Jackson ImmunoResearch Labs Cat# 111-035-003, RRID:AB_2313567)

The primary antibodies used in this study for immunofluorescence are:
anti-HA (1:1000, Cell Signaling Technology Cat# 3724, RRID:AB_1549585)
anti-mHSP60 (1:1000, Abcam Cat# ab128567, RRID:AB_11145464)
anti-mHSP60 (1:500, Novus Biologicals Cat#NBP3-05536, RRID:AB_3086708)
anti-GFP (1:250-1:500, Thermo Fisher, Cat# A10262; RRID:AB_2534023)
anti-WIPI2 (1:500, Abcam, Cat# ab105459; RRID: AB_10860881)
anti-B17.2L (a kind gift from Prof. Mike Ryan (Monash University) and previously generated in rabbits using recombinant full length B17.2L antigen)
anti-TBK1 (1:200, Cell Signaling Technology Cat# 38066, RRID:AB_2827657)
anti-ATG13 (1:200, Cell Signaling Technology, Cat# 13468; RRID: AB_2797419)

The secondary antibodies for immunofluorescence used in this study are:
AlexaFluor-488 goat anti-rabbit IgG (H+L) (1:250-500, Thermo Fisher, Cat# A11008; RRID: AB_143165)
AlexaFluor-555 goat anti-rabbit IgG (H+L) (1:250-500, Thermo Fisher, Cat# A21428; RRID: AB_2535849)
AlexaFluor-647 goat anti-rabbit IgG (H+L) (1:250-500, Thermo Fisher, Cat# A21244; RRID: AB_2535812)
AlexaFluor-488 goat anti-mouse IgG (H+L) (1:250-500, Thermo Fisher, Cat# A21202; RRID: AB_141607)
AlexaFluor-555 goat anti-mouse IgG (H+L) (1:250-500, Thermo Fisher, Cat# A21422; RRID: AB_2535844)
AlexaFluor-647 goat anti-mouse IgG (H+L) (1:250-500, Thermo Fisher, Cat# A21235; RRID: AB_2535804)
AlexaFluor-488 goat anti-chicken IgY (H+L) (1:250-1:500, Thermo Fisher, Cat# A32931, RRID: AB_2762843)
AlexaFluor-647 goat anti-chicken IgY (H+L) (1:250-1:500, Thermo Fisher, Cat# A21449; RRID: AB_2535866)

Validation

Antibodies were selected based on their use in other publications and/or validation by the manufacturers for their respective application. Where possible, knockout cell lines were used to validate the specificity of the antibodies further. These were FIP200, NAP1, OPTN, p62,/SQSTM1, SINTBAD, TBK1, p-TBK1, Parkin, WIPI2, ATG13, and ULK1. Alternatively, some antibodies were validated by verifying their known localization when we separated cytosol from mitochondria in cell fractionation experiments, allowing us to verify the specificity of antibodies based on the cellular localization of the target protein, such as our loading controls mHSP60, B-Actin, a-Tubulin, COXII. Antibodies against affinity tags were validated by comparing overexpression versus non-transfected cell lines (anti-HA). The phospho-Ub antibody was validated by its known upregulation upon O/A treatment of cells and this was confirmed to lead to an increased signal, in line with what is found in literature (PMID: 32142685). The anti-B17.2L was previously published and validated (PMID: 30679426). Specificity of secondary antibodies was validated by omitting primary antibodies.

Eukaryotic cell lines

Policy information about cell lines and Sex and Gender in Research

Cell line source(s)	All parental cell lines (HeLa, HEK293T, HEK293F) were acquired from the American Type Culture Collection (ATCC). HAP1 cells (RRID:CVCL_Y019) were acquired from Horizon Discovery. HAP1 knockout lines ATG5 (RRID:CVCL_SE00) and FIP200/RB1CC1 (RRID:CVCL_TI59) were acquired from Horizon Discovery. HeLa knockout cell lines were generated during this study and submitted to Cellosaurus. Sf9 insect cells were acquired from Thermo Fisher (12659017, RRID:CVCL_0549).
Authentication	Authentication was performed upon first arrival in the lab based on morphology and karyotyping.
Mycoplasma contamination	All cell lines were routinely tested for mycoplasma contamination. All cell lines were negative throughout the study.
Commonly misidentified lines (See ICLAC register)	The cell lines used in this study are not listed as commonly misidentified cell lines. This was verified in the ICLAC table of commonly misidentified cell lines.

Flow Cytometry

Plots

Confirm that:

- [x] The axis labels state the marker and fluorochrome used (e.g. CD4-FITC).
- [x] The axis scales are clearly visible. Include numbers along axes only for bottom left plot of group (a 'group' is an analysis of identical markers).
- [x] All plots are contour plots with outliers or pseudocolor plots.
- [x] A numerical value for number of cells or percentage (with statistics) is provided.

Methodology

Sample preparation	HeLa cells were transduced with lentiviral or retroviral vectors that would express the fluorophore. Cells were treated according the experimental protocol and then collected by removing the medium, washing the cells with 1x PBS (14190169, Thermo Fisher), trypsinization (T3924, Sigma), and resuspending in complete DMEM medium (41966052, Thermo Fisher). Filtered through 35 µm cell-strainer caps (352235, Falcon) and analyzed by an LSR Fortessa Cell Analyzer (BD Biosciences). Lysosomal mt-mKeima was measured using dual excitation ratiometric pH measurements at 405 (pH 7) and 561 (pH 4) nm lasers with 710/50-nm and 610/20-nm detection filters, respectively. Additional channels used for fluorescence compensation were BFP and GFP. Single fluorescence vector expressing cells were prepared to adjust photomultiplier tube voltages to make sure the signal was within detection limits, and to calculate the compensation matrix in BD FACSDiva Software. Depending on the experiment, we gated for BFP-positive, GFP-positive, and mKeima-positive cells with the appropriate compensation. For each sample, 10,000 mKeima-positive events were collected, and data were analyzed in FlowJo (version 10.9.0).
Instrument	LSR Fortessa Cell Analyzer (BD Biosciences)
Software	BD FACSDiva software during data collection and FlowJo10 software (Tree Star Inc., Ashland, OR, USA) for data analysis.
Cell population abundance	Cells were only included when they were viable, single cells (exclusion doublets), and depending on the experiment whether they were GFP-, BFP-, and mt-mKeima positive.
Gating strategy	Gating was optimized, depending on the experiment, for GFP- and/or BFP- and mt-mKeima positive cells after viable singlets were separated from potentially dead cells or doublets based on scatter.

- [x] Tick this box to confirm that a figure exemplifying the gating strategy is provided in the Supplementary Information.
