# Supplementary figures and images for: Control of mitophagy initiation and progression by the TBK1 adaptors NAP1 and SINTBAD
Source: Nat Struct Mol Biol. 2024 Jun 25;31(11):1717–31. doi: 10.1038/s41594-024-01338-y (PMC11564117; doi:10.1038/s41594-024-01338-y)

Figure 1C

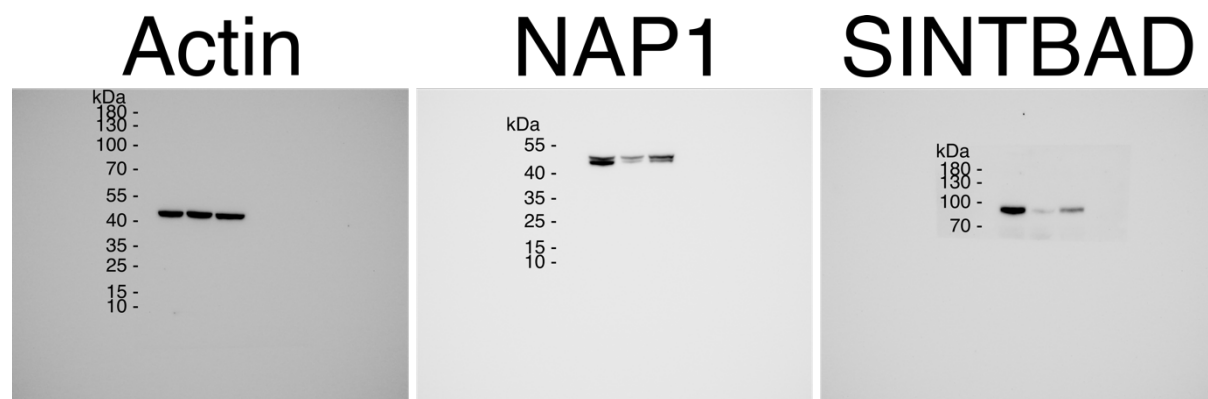

Supplement: Supplementary file 5 — Unprocessed western blots [file 41594_2024_1338_MOESM5_ESM.pdf]

Figure 2D

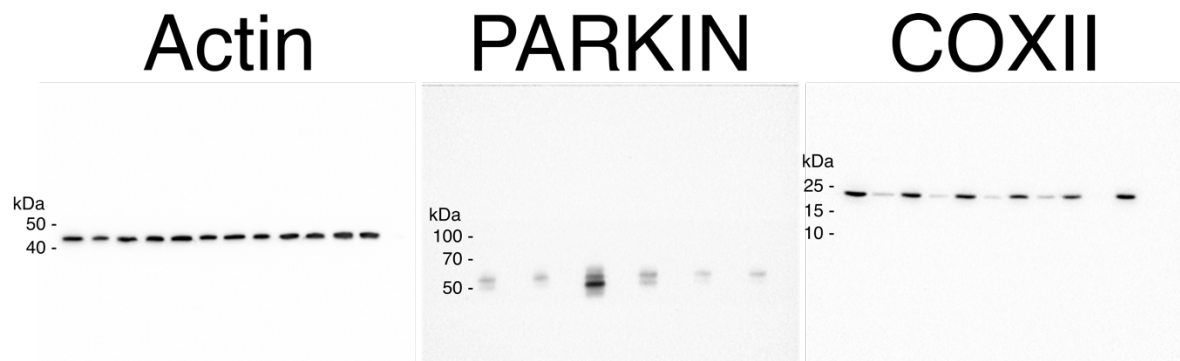

Figure 2E

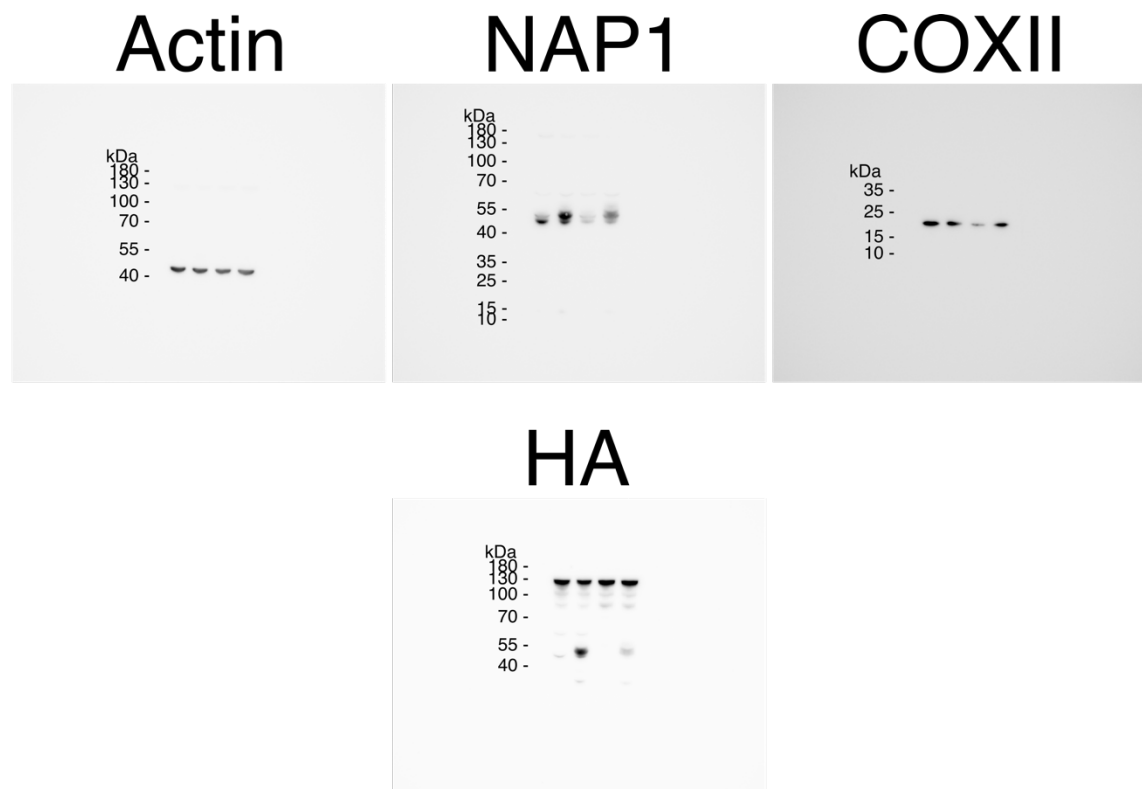

Supplement: Supplementary file 7 — Unprocessed western blots [file 41594_2024_1338_MOESM7_ESM.pdf]

Figure 3A

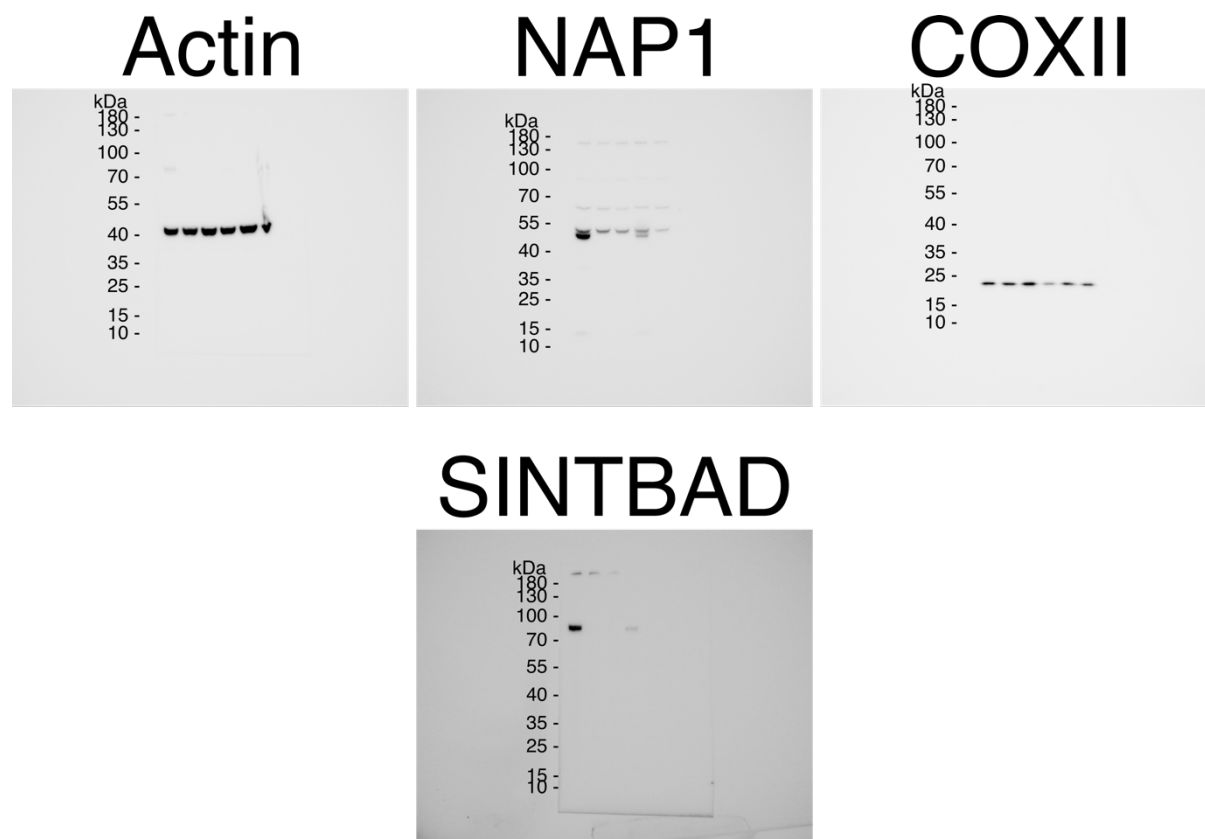

Figure 3C

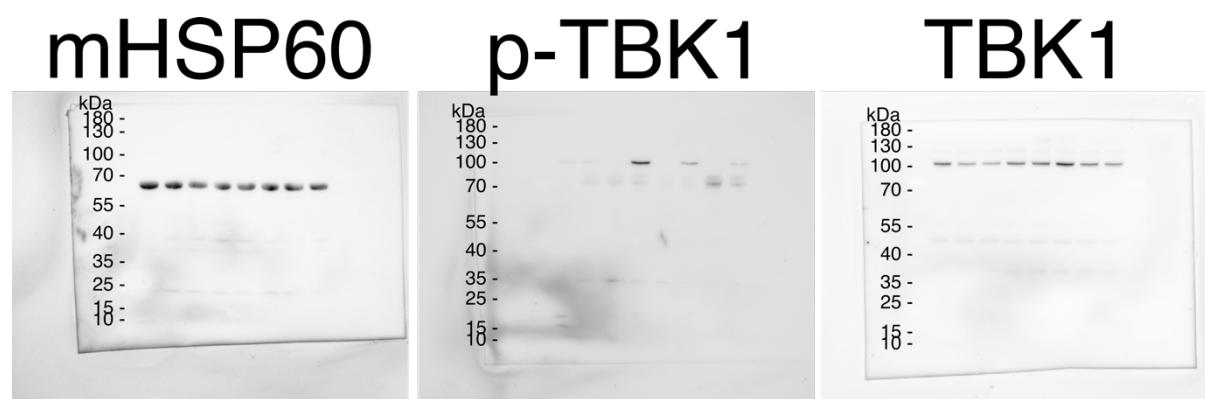

Figure 3E

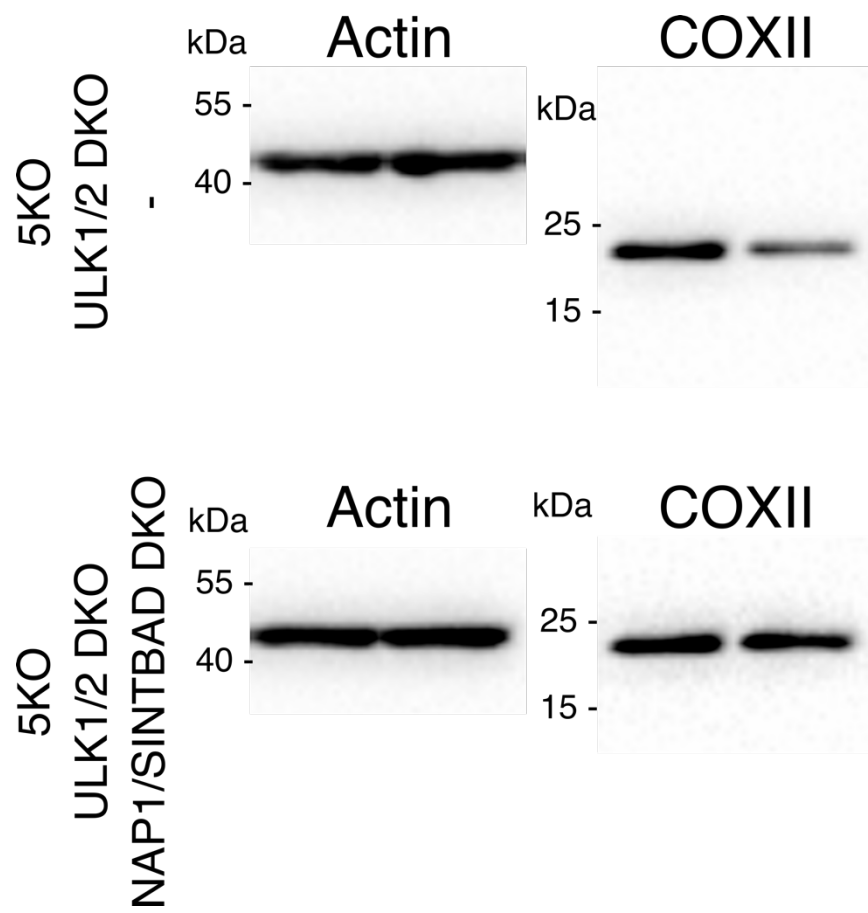

Supplement: Supplementary file 9 — Unprocessed western blots [file 41594_2024_1338_MOESM9_ESM.pdf]

Figure 4F

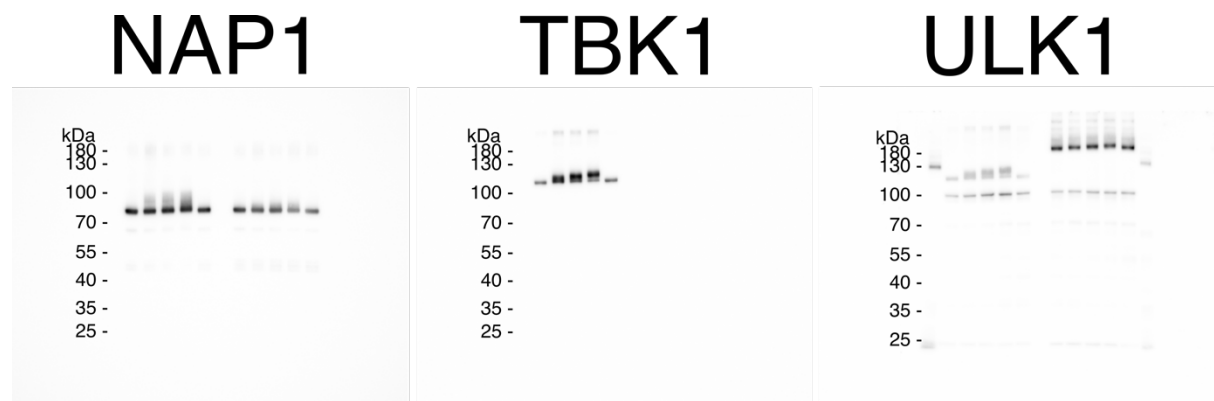

Figure 4H

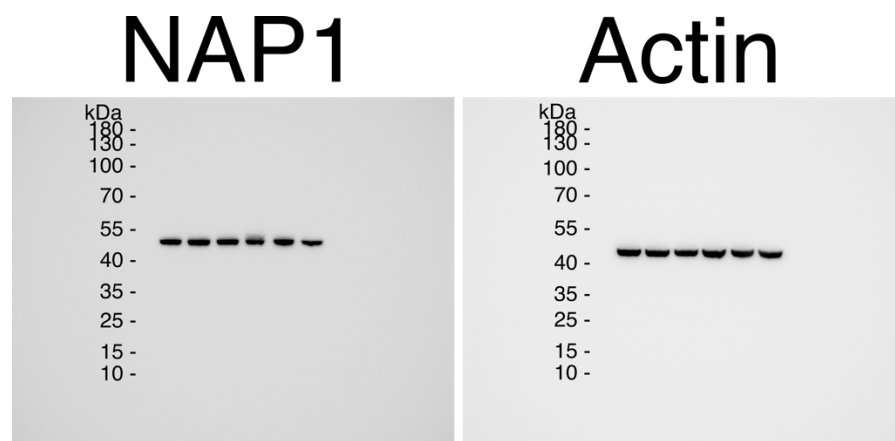

Supplement: Supplementary file 11 — Unprocessed western blots [file 41594_2024_1338_MOESM11_ESM.pdf]

Figure 5C

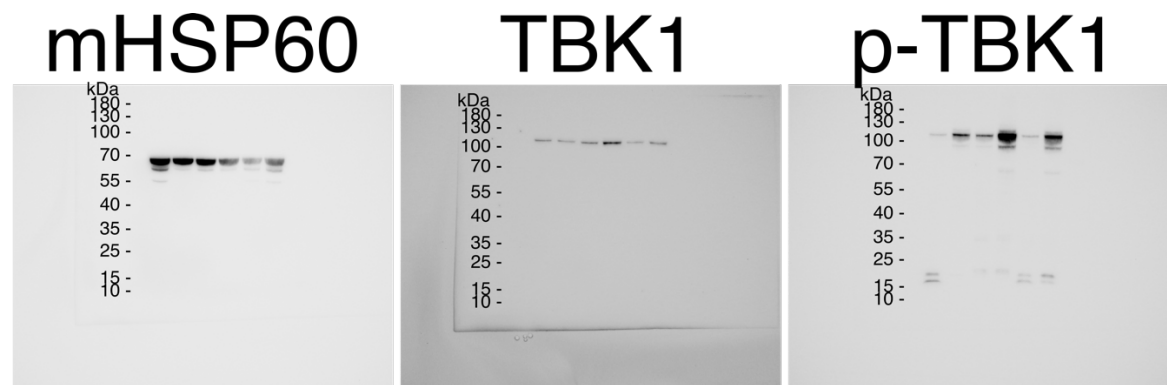

Figure 5D

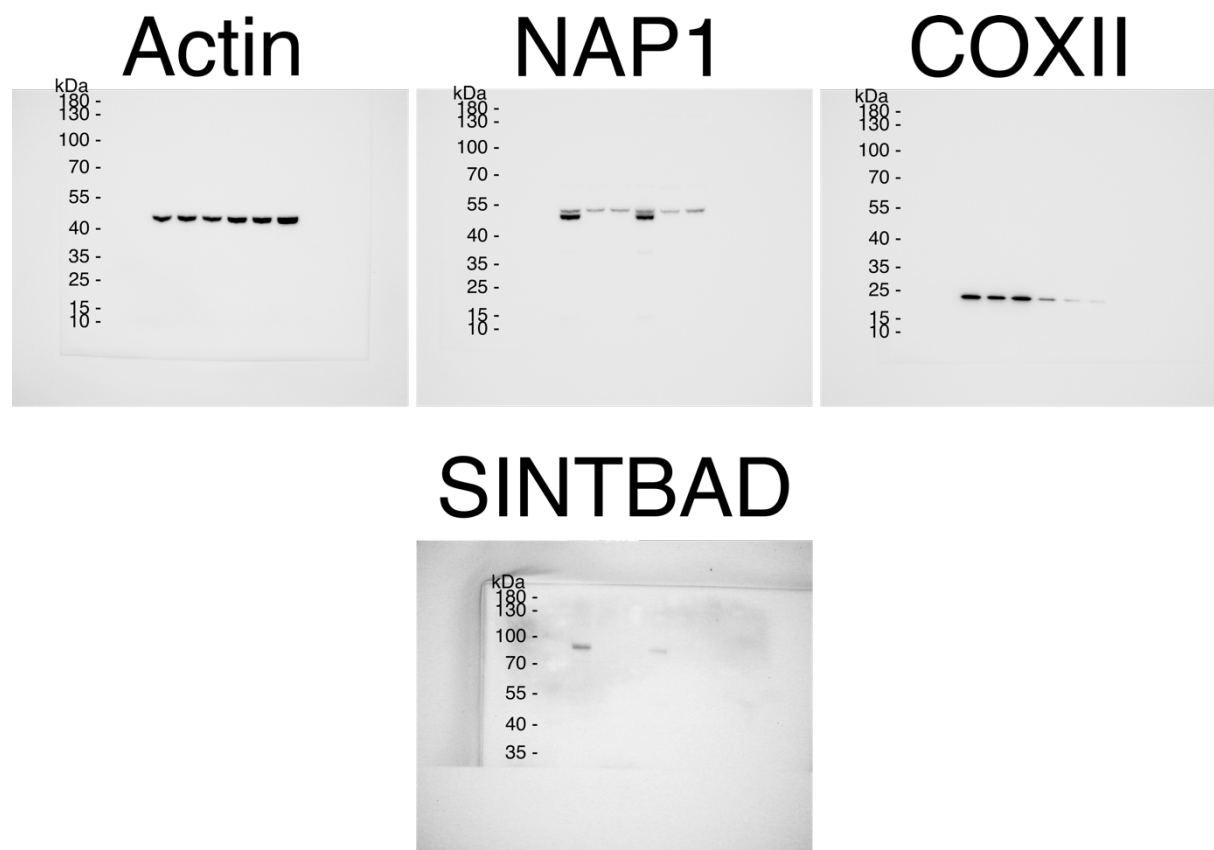

Figure 5E

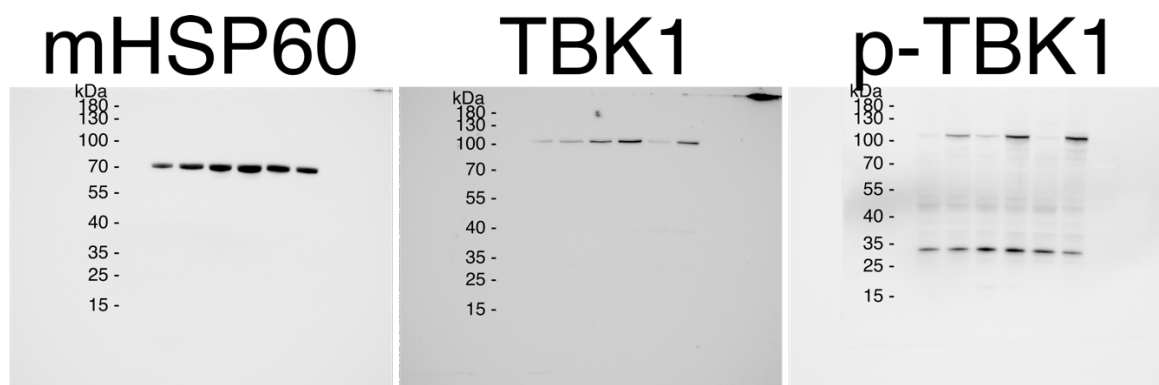

Figure 5F

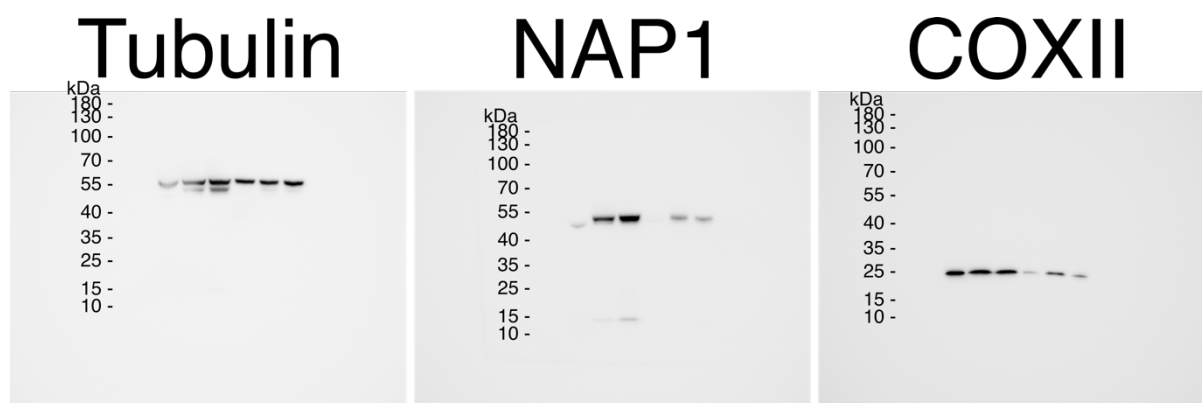

Supplement: Supplementary file 13 — Unprocessed western blots [file 41594_2024_1338_MOESM13_ESM.pdf]

Figure 6A

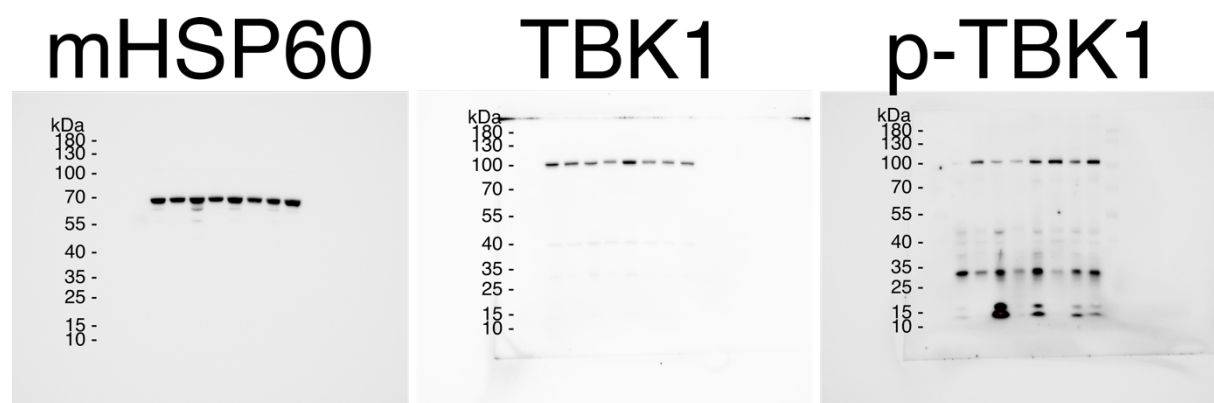

Figure 6B

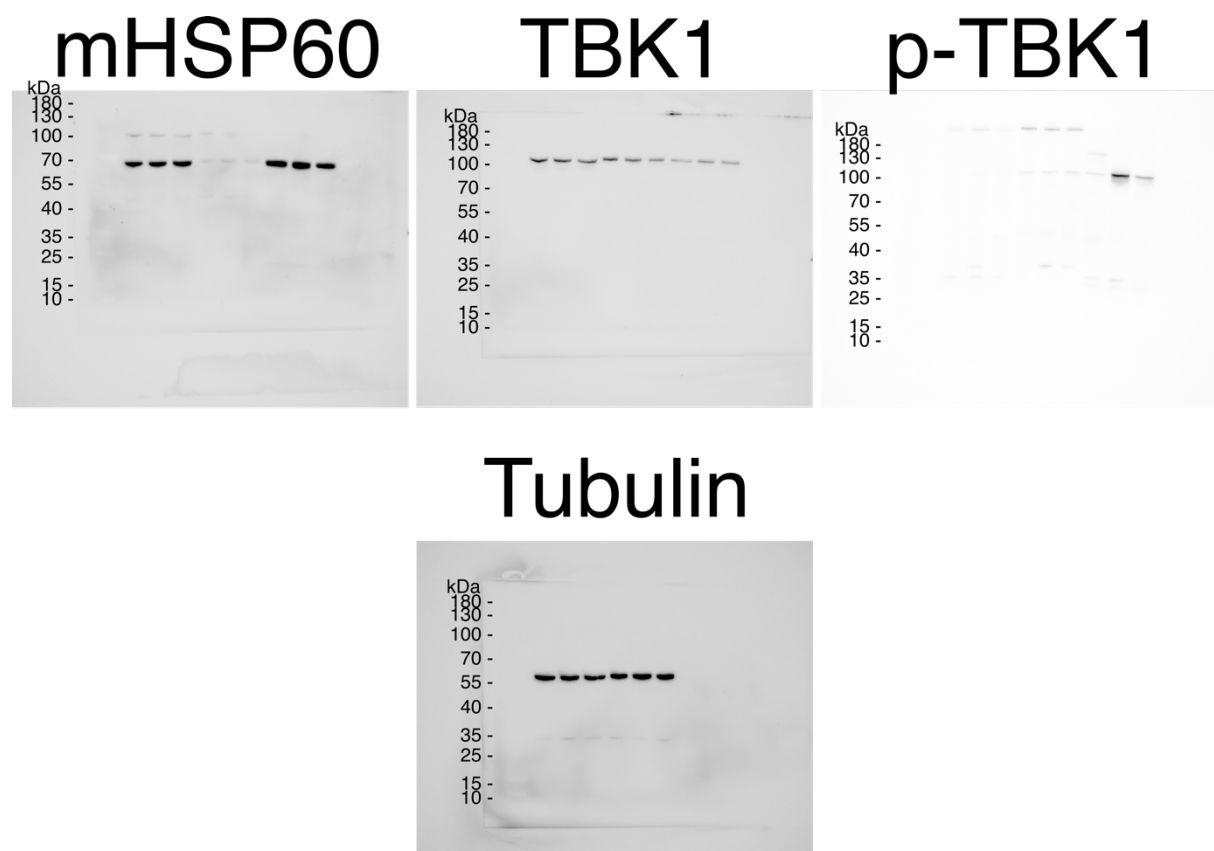

Figure 6C

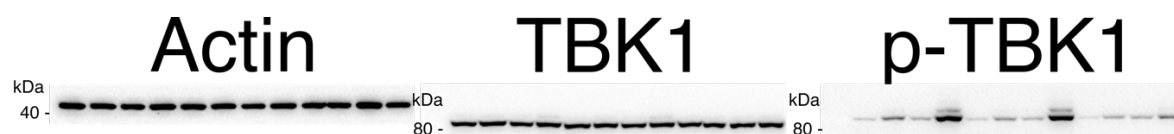

Figure 6F

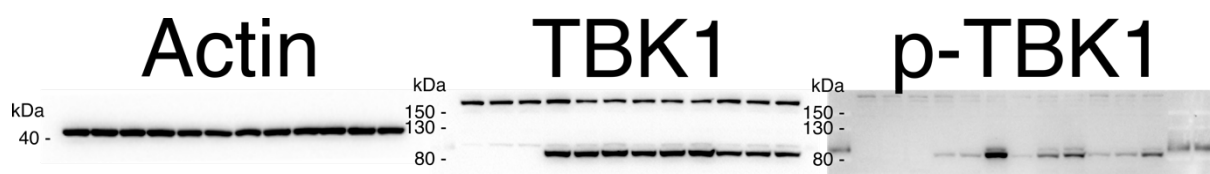

Supplement: Supplementary file 15 — Unprocessed western blots [file 41594_2024_1338_MOESM15_ESM.pdf]

Figure 7B

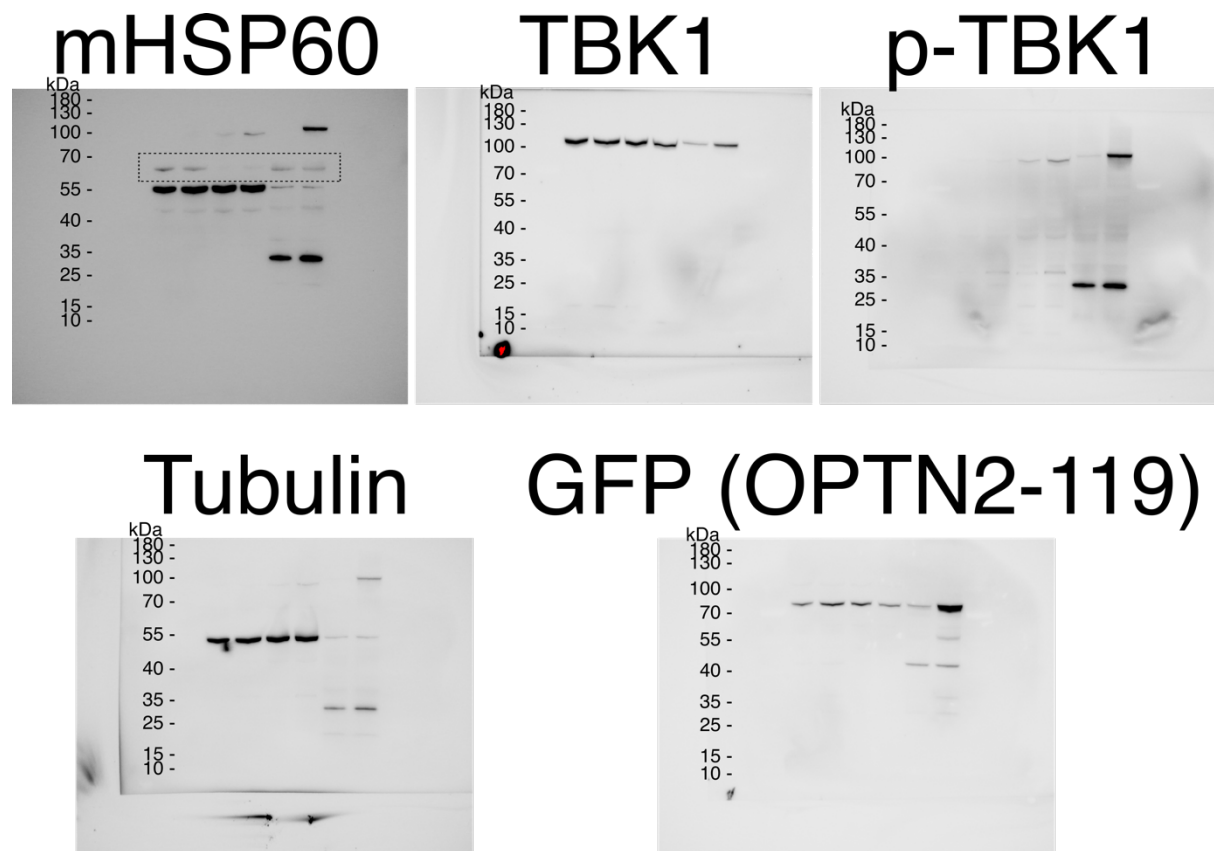

Figure 7E

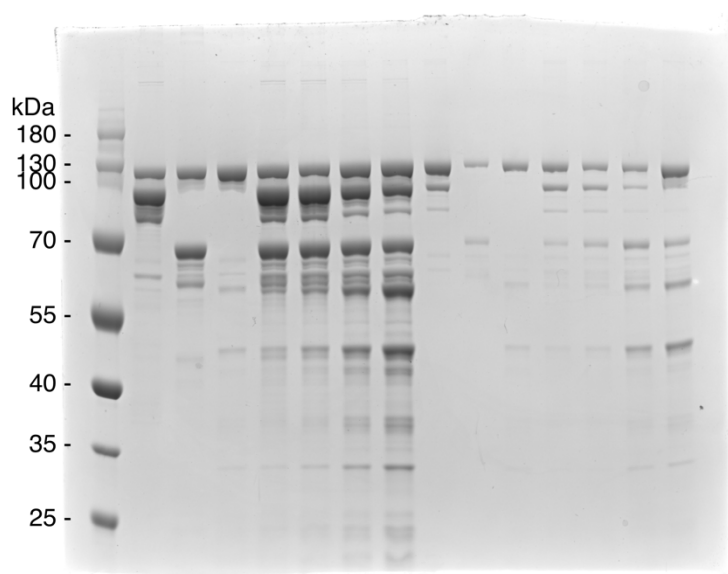

Supplement: Supplementary file 17 — Unprocessed western blots and gels [file 41594_2024_1338_MOESM17_ESM.pdf]

Extended Data Figure 1

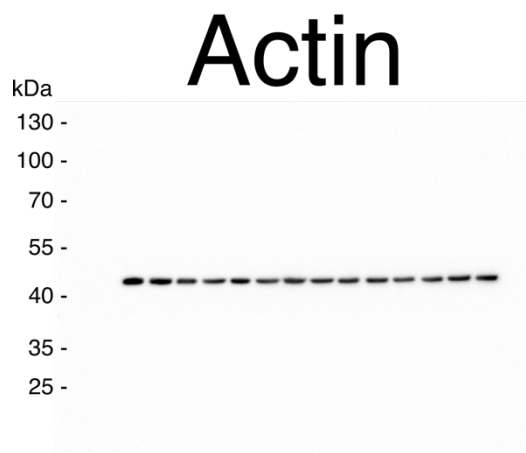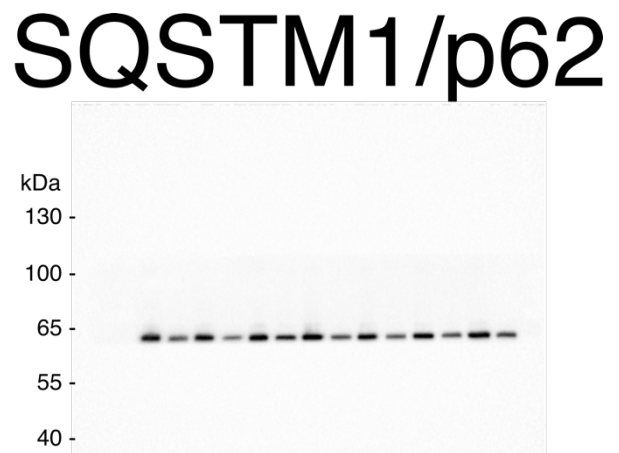

Supplement: Supplementary file 18 — Unprocessed western blots [file 41594_2024_1338_MOESM18_ESM.pdf]

Extended Data Figure 4A

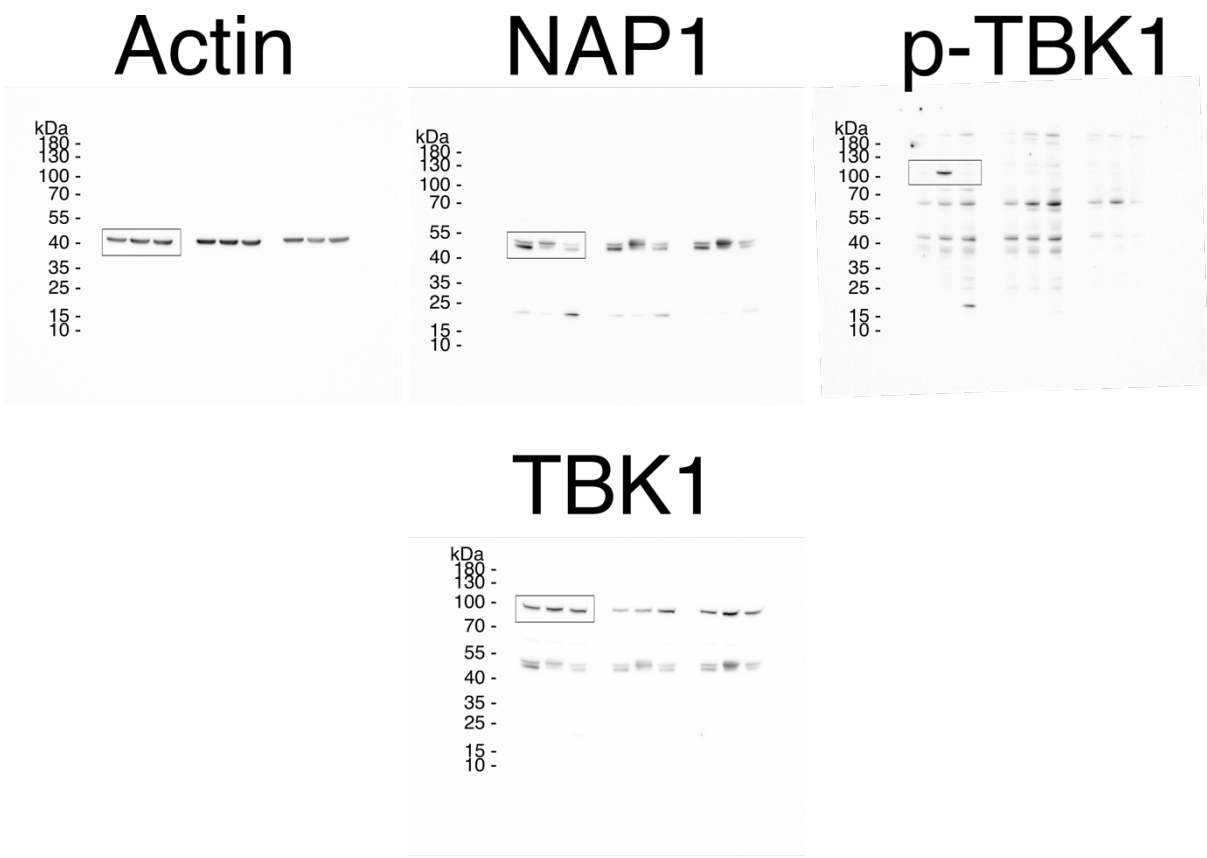

Extended Data Figure 4B

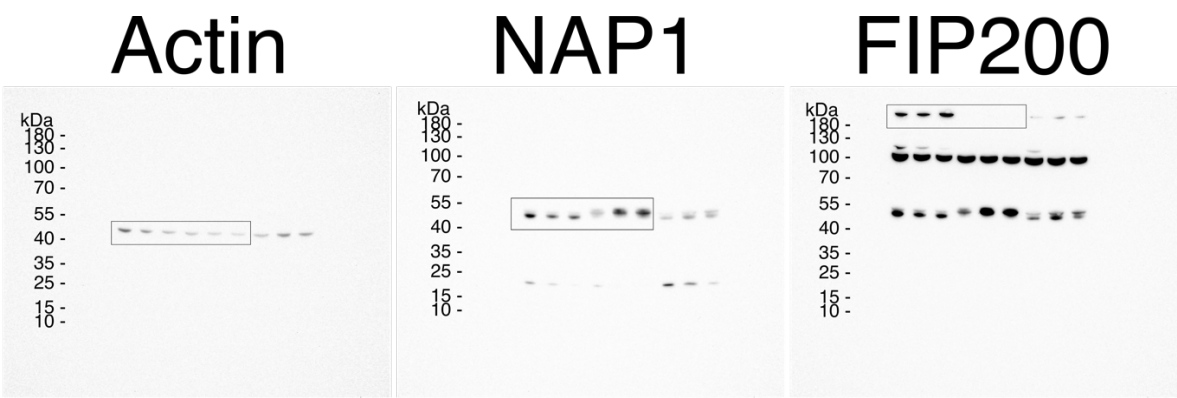

Extended Data Figure 4E

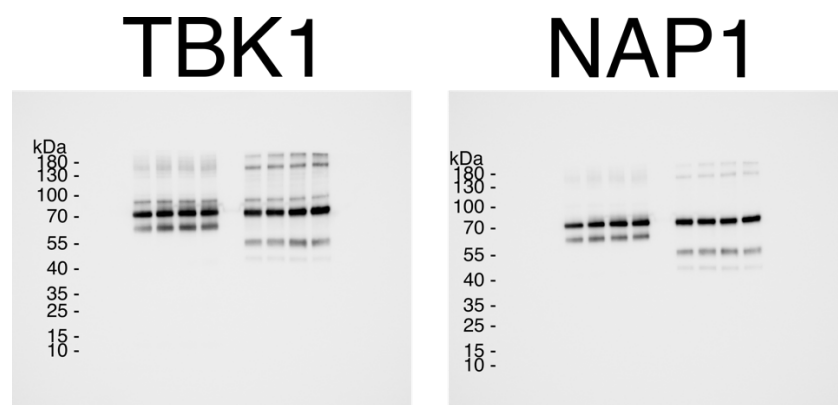

Extended Data Figure 4F

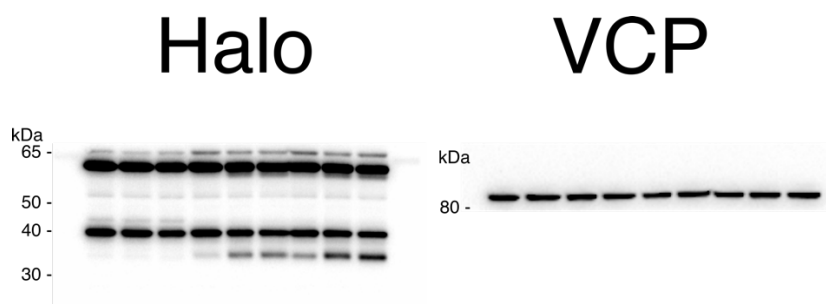

Supplement: Supplementary file 22 — Unprocessed western blots [file 41594_2024_1338_MOESM22_ESM.pdf]

Extended Data Figure 5

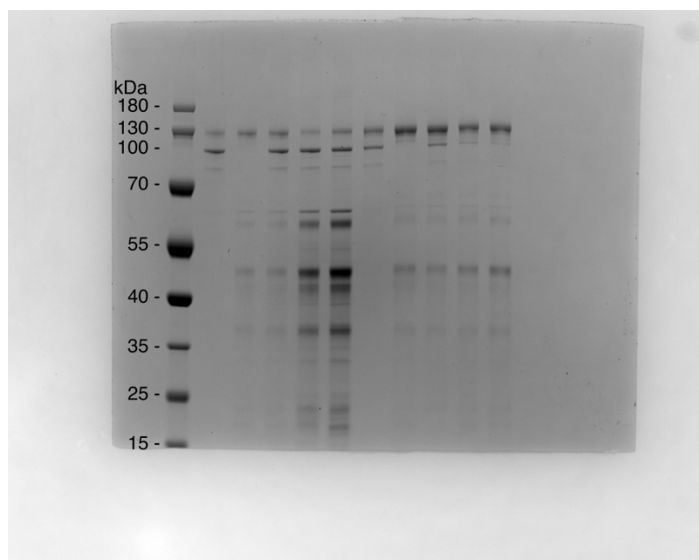

Supplement: Supplementary file 24 — Unprocessed gels [file 41594_2024_1338_MOESM24_ESM.pdf]
