# Supplementary material for: Control of mitophagy initiation and progression by the TBK1 adaptors NAP1 and SINTBAD
Source: Nat Struct Mol Biol. 2024 Jun 25;31(11):1717–31. doi: 10.1038/s41594-024-01338-y (PMC11564117; doi:10.1038/s41594-024-01338-y)
Supplement: Supplementary file 1 — Supplementary Fig. 1 and Supplementary Table 1 [file 41594_2024_1338_MOESM1_ESM.pdf]

nature structural & molecular biology

Supplementary information https://doi.org/10.1038/s41594-024-01338-y

Control of mitophagy initiation and progression by the TBK1 adaptors NAP1 and SINTBAD

In the format provided by the authors and unedited

Supplementary information

FACS Gating strategy for O/A induced mitophagy

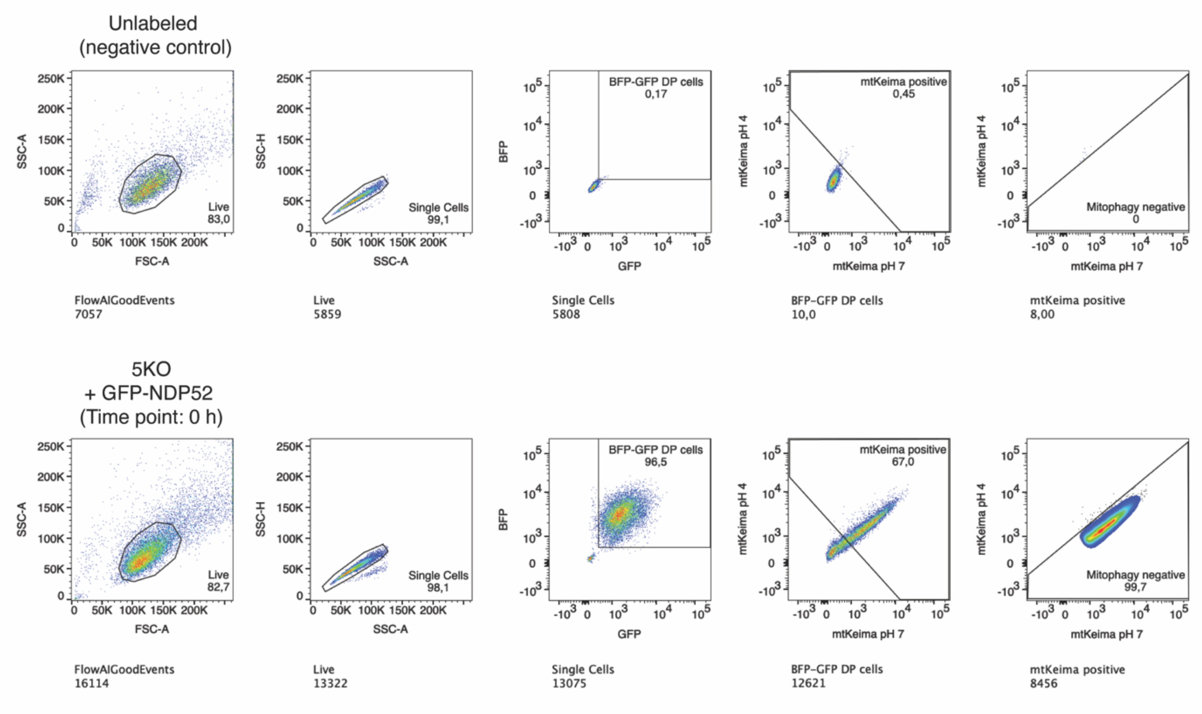

FACS Gating strategy for Rapalog induced mitophagy

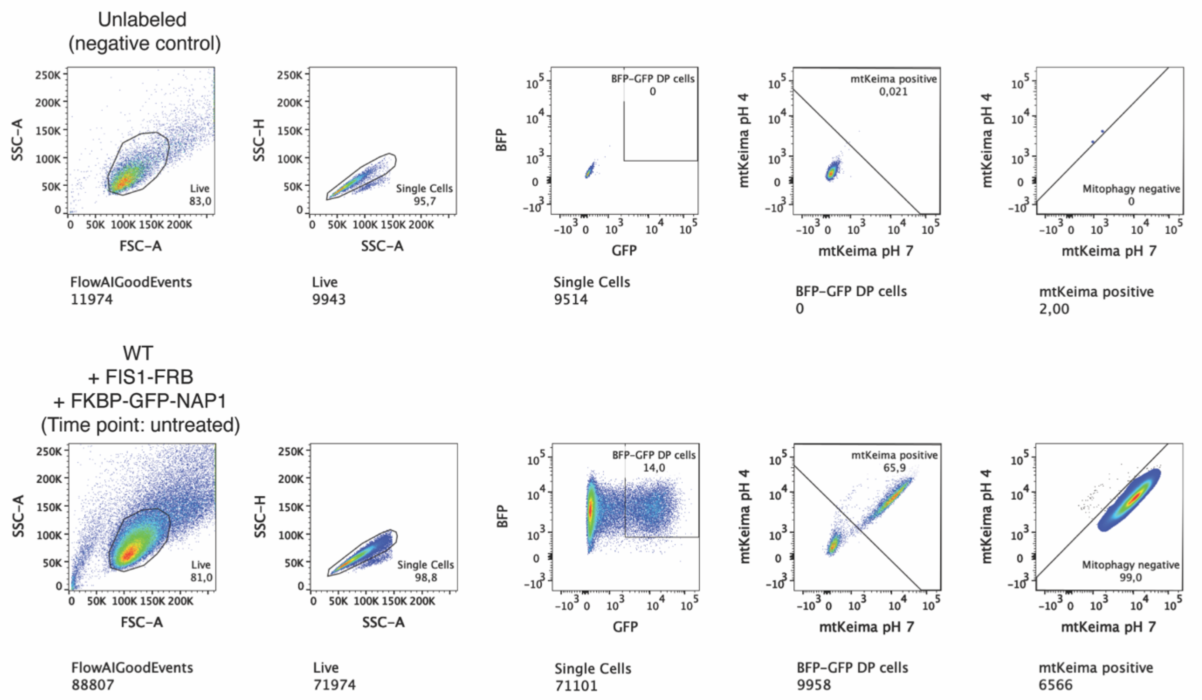

Supplementary Figure 1

Gating strategy for FACS experiments where cells were either treated with O/A or Rapalog. Unstained negative controls are shown. BFP-GFP DP cells represent BFP and GFP double positive (DP) cells. The numbers below each gate represent the number of cells that were gated from the previous plot into the current plot. For instance, 8456 mtKeima positive cells means that a total of 8456 cells are displayed on the mtKeima plot of which only a fraction (indicated in the figures of the main manuscript) are mtKeima positive.

Supplementary Table 1: Details of CRISPR /Cas9-edited cell lines generated this study.

Gene Symbol	Uniprot	GeneID / Location	Targeting strategy	CRISPR gRNA (PAM)	Clone number	Potential unique Alleles	Mutation	Protein impact
***SINTBAD* KO**								
SINTBAD/ TBKBP1	A7MCY6	9755/NC_000017.11	Exon 1	AGTCCCGGAGACCCCTCGCTTGG	#7	3	c.[92_95del];[93_94insG];[~200bp del, ~200bp ins]	p.[S31Lfs*46];[L32AFS*49];[frameshifting and introducing stop codon]
***NAP1* KO**								
NAP1/AZI2	Q9H6S1	64343/NC_000003.12	Exon 6	CAAATGACAGGATCCATATCAGG	#32	3	c.[588_640del];[591_602del];[588_640del]	p.[D197Ffs*2];[D197_Q200del];[D197Ffs*2]
					#62	3	c.[589_592del];[588_595del]; [599-176_644del]	p.[D197Hfs*6];[D197Sfs*17];[D197Kfs*1]
***NAP1/SINTBAD* DKO**								
NAP1/AZI2	Q9H6S1	64343/NC_000003.12	Exon 6	CAAATGACAGGATCCATATCAGG	#14 (made from NAP1 KO #62)	3	c.[589_592del];[588_595del]; [599-176_644del]	p.[D197Hfs*6];[D197Sfs*17];[D197Kfs*1]
SINTBAD/ TBKBP1	A7MCY6	9755/NC_000017.11	Exon 1	AGTCCCGGAGACCCCTCGCTTGG	#14 (made from NAP1 KO #62)	2	c.[92_96del];[92_102del]	p.[S31Wfs*48];[S31*]
***NAP1/SINTBAD DKO*/penta KO**								
NAP1/AZI2	Q9H6S1	64343/NC_000003.12	Exon 6	CAAATGACAGGATCCATATCAGG	#20	11	c.[595_596ins1bp]/[595_596ins5bp]/[595_596ins20bp];[576_610del];[586_607del]; [576_614del];[576_609del];[579_615del];[582_609del];[587_609del];[595_596del]	p.[frameshifting and introducing stop codon];[K192Nfs*13]; [T196Ifs*1];[A193_K205del];[K192Nfs*1];[K194Afs*6];[K194Nfs*1];[D197Efs*12];[Y199Sfs*17]
					#26	1	c.[595_596ins1bp]	p.[frameshifting and introducing stop codon]
SINTBAD/ TBKBP1	A7MCY6	9755/NC_000017.11	Exon 1	AGTCCCGGAGACCCCTCGCTTGG	#20	1	c.[92_93ins1bp]	p.[frameshifting and introducing stop codon]
					#26	4	c.[92_93ins1bp];[82_92del];[93_104del]; [92_103del]	p.[frameshifting and introducing stop codon]; [G28_S31del];[L32_D35del];[S31_G34del]
***NAP1/SINTBAD/ULK1/2* 4KO/penta KO**								
NAP1/AZI2	Q9H6S1	64343/NC_000003.12	Exon 6	CAAATGACAGGATCCATATCAGG	#27	5	c.[595_596ins8bp];[596_636delins47bp]; [596_636delins43bp];[595_636delins29bp];[596_636delins30]	p.[frameshifting and introducing stop codon]; [199_212delins16a.a];[Y199*];[Y199Sfs*23];[199_212delins16a.a];[Y199*]
SINTBAD/ TBKBP1	A7MCY6	9755/NC_000017.11	Exon 1	AGTCCCGGAGACCCCTCGCTTGG	#27	13	c.[92_93ins20bp]/[92_93ins19bp]/[92_93ins11bp];[92_93ins18bp];[78_112del];[87_112del];[89_112del];[91_92del][75_112del];[90_112del];[91_110del];[91_103del];[92_109del]	p.[frameshifting and introducing stop codon];[S31_L32ins6a.a]; [P27Rfs*42];[p30rFS*42];[S31_S38del];[S31Afs*49];[S26Rfs*42];[S31Rfs*42];[S31Lfs42*];[S31Tfs*6];[S31_M36del]

The indels in the indicated knockout cell lines were sequenced by Sanger sequencing and analyzed via Synthego by ICE v2 CRISPR Analysis Tool (synthego.com/products/bioinformatics/crispr-analysis) to detect possible edits and their effects on the translated proteins are presented in

“Mutation” column and “Protein impact” column respectively. The format in these columns was done according to Human Genome Variation Society (HGVS; http://varnomen.hgvs.org/). With genes that have multiple splice variants, the canonical isoform chosen by Uniprot was used to determine mutation positions. del = deletion; ins = insertion; c. = coding DNA; bp = base pair; p. = protein; fs = frame shift; * = stop codon; [?] = affecting splicing; a.a = amino acid. The numbers after the asterisks denote the numbers of amino acids between the first amino acid changed following the mutation(s) and the first subsequent stop codon encountered.
